# Health-education to prevent COVID-19 in schoolchildren: a call to action

**DOI:** 10.1186/s40249-020-00695-2

**Published:** 2020-07-01

**Authors:** Darren J. Gray, Johanna Kurscheid, Mary Lorraine Mationg, Gail M. Williams, Catherine Gordon, Matthew Kelly, Kinley Wangdi, Donald P. McManus

**Affiliations:** 1 Department of Global Health, Research School of Population Health, Australian National University, Canberra, Australia; 2 School of Public Health, University of Queensland, Brisbane, Australia; 3 Infectious Diseases Division, QIMR Berghofer Medical Research Institute, Brisbane, Australia

**Keywords:** COVID-19, Children, Health education, Health promotion, Prevention

## Abstract

**Background:**

There is currently considerable international debate around school closures/openings and the role of children in the transmission of coronavirus disease 2019 (COVID-19). Whilst evidence suggests that children are not impacted by COVID-19 as severely as adults, little is still known about their transmission potential, and with a lot of asymptomatic cases they may be silent transmitters (i.e. infectious without showing clinical signs of disease), albeit at a lower level than adults. In relation to this, it is somewhat concerning that in many countries children are cared for, or are often in close contact with, older individuals such as grandparents ─ the age group most at risk of acquiring serious respiratory complications resulting in death.

**Main text:**

We emphasise that in the absence of a vaccine or an effective therapeutic drug, preventive measures such as good hygiene practices ─ hand washing, cough etiquette, disinfection of surfaces and social distancing represent the major (in fact only) weapons that we have against COVID-19. Accordingly, we stress that there is a pressing need to develop specific COVID-19 prevention messages for schoolchildren.

**Conclusion:**

An entertainment education intervention for schoolchildren systematically implemented in schools would be highly effective and fill this need. With such measures in place there would be greater confidence around the opening of schools.

## Background

The scant current scientific evidence suggests that coronavirus disease 2019 (COVID-19) is less severe in children than in adults and that children are more likely to be asymptomatic or have mild disease [1]. However, these observations provide no elucidation of the potential role of children in transmitting the disease.

Well-documented scientifically is the major transmission role children have had historically in the spread of respiratory infections — generally through their close interactions in schools and child-care centres. Moreover, children and teenagers aged 5–17 are considered to play the most important role in mass influenza A epidemics [2]. While the lack of severity of COVID-19 in children contrasts with that of other respiratory viruses such as influenza, similarities in the mode of transmission still exist. Recent studies have demonstrated severe acute respiratory syndrome coronavirus 2 (SARS-CoV-2) does infect children; with about 50% of pediatric cases asymptomatic [1]. Consequently, they may have an important role in transmission — albeit at lower levels than adults — and could be “silent” transmitters (i.e. infectious without showing clinical signs of disease). Worryingly, in many countries it is common for children to be cared for, or be in close contact with, elderly people, for example, grandparents — the very age group most at risk of acquiring serious respiratory complications resulting in death.

In the absence of a vaccine or effective therapeutic drugs, preventive measures such as: good hygiene practices — hand washing, cough etiquette, disinfection of surfaces and social distancing represent the major weapons against COVID-19. The World Health Organization (WHO) states, “the best way to prevent and slow down transmission is to be well informed about SARS-CoV-2, the disease it causes and how it spreads” [3]. We have seen that health and hygiene campaigns, which reinforce consistent messaging and persuade people to alter their habits, are effective in reducing infection rates. To date, however, most of the messaging has targeted the general population — not children specifically. Children are able to copy parent behaviour, but are not equipped with a true understanding of why they are being asked to make changes, potentially resulting in confusion, fear and lapses in hygiene or social protocols. Development of an appropriate and engaging hygiene and social distancing education campaign targeting children is urgently needed in order to reinforce adult messages appropriately and maximise child compliance. Accordingly, we provide an overview of COVID-19-related health education/promotion messaging currently available, and posit the way forward for engaging schoolchildren.

## Main text

The WHO is the main international body providing information to the public, health sector, and governments. Messaging around COVID-19 for the public incorporates: global situation updates; information on transmission; signs and symptoms of infection; prevention and control practices (i.e. good hygiene and physical distancing); and specific information for pregnant women and travelers, or those recently visiting or returning from hot spot areas. Resources for the media also explain how to relay messages regarding prevention measures. Delivery of these messages uses a variety of formats including information sheets, videos and infographics. These are available on the WHO website and their social media platforms. Advice is provided for parents on communicating with children about COVID-19. Some materials are available for schools on age-appropriate health education regarding the virus and the associated disease but messages specifically targeting children are lacking and have caused confusion and anxiety for those too young to engage with the current campaigns.

Messages similar to those provided by WHO, but with a local context, have been disseminated by the United States Centers for Disease Control (US CDC), which has a series of COVID-19 educational videos available on their website and on YouTube. In countries outside the USA, departments and ministries of health have also released similar messaging campaigns in print and multimedia formats via mass media channels and social media platforms. Specific resources are available for health professionals, including aged care providers, pathology providers and health care managers; and while there is a smattering of messages targeting young children — mainly via YouTube — there is a serious deficiency of appropriate specific resources systematically embedded in schools to provide consistent messaging for children worldwide.

Health messages that are positive, engaging, entertaining, fun and humorous, while providing accurate age-appropriate understanding are important features when targeting schoolchildren. The value of a moving image in health education was highlighted as early as 1988 in a manual published by the WHO [4], which pointed out that no other media, creates such lively interest as television. Television programs such as Sesame Street, Between the Lions, and Blue’s Clues have contributed to reinforcing positive influences in the cognitive development of young children. Cartoons also have a long history of popularity with children — highlighted by the Disney and Warner Brothers franchises — and are used to reinforce learning and interaction. As such, this entertainment-education approach has provided a highly effective forum for health education interventions targeting schoolchildren. Videos/cartoons have proven to be of great value [4]. Compared to text-based teaching videos/cartoons can reinforce desired behaviour for children as they learn through direct observation, a critical element in behaviour [5].

With the urgent need to develop specific COVID-19 prevention messages for schoolchildren it is logical to develop a cartoon video-based entertainment education approach, with a discrete, engaging and highly informative story line, emphasising correct hand washing procedures and the social distancing concept. One highly successful and proven intervention is “The Magic Glasses”, an inexpensive and engaging 12-min cartoon-based hygiene education intervention package, which is combined with classroom discussions, drawing and essay competitions, and a pamphlet (derived from the cartoon) to reinforce the health messages. The cartoon can be accessed at https://www.nejm.org/doi/full/10.1056/nejmoa1204885. The focus of the intervention is to visually inform children about the transmission and prevention of soil-transmitted helminths; it has been rigorously tested with success in resource-poor settings across Asia including China [6], the Philippines and Vietnam; and is currently being developed for the Lower Mekong Region including its adaptation to carcinogenic liver fluke infections. To our knowledge, no such cartoon-based intervention is currently available as a preventive approach against COVID-19.

The cartoon concept enables children to identify with characters to visualise the intestinal parasitic worms and their eggs in people and the environment to reinforce the importance of good hygiene and associated health behaviours [5, 6]. This is directly applicable to the transmission dynamics of SARS-CoV-2 — whereby virus would be visualised in people and the environment — and the associated messages for prevention. The formative research process used to develop the original “Magic Glasses” [7] would be used here to identify risk factors and drivers for behaviour change in order to translate them into the preventive messages. Key messages of such an intervention could include hand washing, care in coughing and sneezing, tissue use and disposal, physical distancing (which particularly lends itself to visual display) and what to do when feeling unwell. It also reinforces how the virus behaves to assist children’s understanding and allay fear. Readily available for systematic delivery in schools — implemented as part of the curriculum over the course of the school year — and for parents, the cartoon would be accessible via mainstream media and social media platforms (e.g. YouTube). The cartoon would also be applicable for television broadcast. Furthermore, and very importantly, utilising a scientifically validated health education intervention to disseminate COVID-19 messaging to children ensures that reliable and factual information is delivered through official channels (such as schools) to an audience who are considered to be highly vulnerable to becoming victims of misinformation or ‘infodemics’ [8].

## Conclusions

Health education and promotion are important components of disease prevention activities in general, but during disease outbreaks and health emergencies, they play a key role in an active response by offering well-established tools (especially important in the absence of specific drug therapies and vaccines) to communicate and engage quickly and effectively with the public and prevent infections. Messaging specifically targeting children who may well be acting as “silent” transmitters of COVID-19 is presently lacking. A video/cartoon-based entertainment-education intervention would fill this need. This is important now during the peak of transmission and, most importantly, to reinforce and habituate good hygiene practices long-term to prevent rebound infections, given the pandemic is expected to continue for at least the remainder of 2020 if not longer if COVID-19 becomes endemic. Targeted COVID-19 messages to children will also be effective for influenza prevention and considerably help reduce COVID-19/Influenza co-morbidity; in addition, it will provide a targeted explanation to help minimise fear and anxiety in young children. With the current international debate around school closures/openings, having this preventive intervention in place in schools would contribute to mitigating the risk and provide greater confidence for schools to be open to authorities and parents alike.

## Data Availability

N/A

## Abbreviations

WHO: World Health Organization
COVID-19: Coronavirus disease 2019
SARS-COV-2: Severe acute respiratory syndrome coronavirus-2
US CDC: United States Centers for Disease Control
